# Supplementary material for: Comparative efficacy of cisternal drainage versus external ventricular drainage in severe aneurysmal subarachnoid hemorrhage
Source: PLoS One. 2026 Jan 7;21(1):e0340013. doi: 10.1371/journal.pone.0340013 (PMC12779065; doi:10.1371/journal.pone.0340013)
Supplement: Attachment — Submitted filename: Response to Reviewers.docx [file pone.0340013.s004.docx]

**Response to Reviewer 1：**

We would like to express our sincere appreciation for taking time from your busy schedule to review our manuscript and provide us with your valuable comments. We will address each of your questions point by point, and we hope our responses will meet your expectations and resolve any concerns you may have.

**Question 1**:

Through a pterional approach, following aneurysm clipping, the optico-carotid cistern was exposed, and a soft drainage catheter was placed within this cistern. The purpose is to continuously drain bloody cerebrospinal fluid in the early postoperative period, aiming to reduce the risk of cerebral vasospasm, alleviate meningeal irritation caused by bloody CSF and monitor dynamic changes in intracranial pressure. This differs fundamentally in both function and location from an external ventricular drain placed within the ventricles or a subcutaneous drainage catheter. It acts directly on the subarachnoid space in the surgical area.

Regarding fenestration of the lamina terminalis, we thank the reviewer for pointing out this omission in reporting. Lamina terminalis fenestration is a routine step at our center during craniotomy for anterior circulation aneurysms. Based on our review of surgical records, among the 31 patients included in this study who underwent surgical clipping, all patients concurrently underwent lamina terminalis fenestration.

We have added the precise definitions and this information to the **Interventions** and **Cisternal drain technique** section of the revised manuscript to avoid any ambiguity.

**Question 2:**

We thank the reviewer for raising this critical point. The choice between EVD and EVD+CD at our institution was indeed influenced by a combination of factors, including the treating physician's preference and their interpretation of the patient's clinical and radiological status, rather than a strict randomized protocol.

However, to address the potential bias introduced by this non-randomized allocation, we have taken the following steps:

1. **Baseline Characteristics Comparison:** As presented in table 1, we conducted a thorough comparison of baseline characteristics (e.g., age, GCS at admission, Hunt-Hess score, aneurysm location, etc.) between the EVD and EVD+CD groups. We found no statistically significant differences in these key prognostic variables, suggesting that the two groups were largely comparable at baseline.

2. **Statistical Adjustment:** Furthermore, we performed Binary logistic regression analysis to adjust for potential confounders (such as gender, age, GCS, Hunt-Hess, hospitalization and GOS). Even after adjustment, the association between hospitalization and different groups remained significant (*p*=0.030).

We acknowledge that residual confounding cannot be entirely ruled out in an observational study. We have added a sentence in the **Discussion section** to explicitly state this limitation. Nonetheless, we believe our analysis provides valuable real-world evidence on the comparative effectiveness of these two procedures.

**Question 3:**

We sincerely thank you for raising this critical point. We fully understand your concern that excluding patients with early mortality may introduce "survivorship bias." Please allow us to explain the rationale behind this exclusion criterion in our study design and provide supplementary analyses to address your valid concerns.

1. The primary aim of this study was to evaluate the long-term functional recovery and its influencing factors specifically in aSAH patients who survived the acute postoperative phase and had complete follow-up data. Including patients with a postoperative survival of less than 6 months (who typically succumb to early clinical deterioration) and those lost to follow-up would fundamentally confound the interpretation of this specific objective. For instance, factors predicting "good functional recovery" are often distinct from those predicting "short-term survival." Combining these fundamentally different outcomes in a single analytical model would render the model invalid and lead to ambiguous, unreliable conclusions.
2. We reassigned all patients with postoperative survival <6 months and those lost to follow-up to the worst outcome category (GOS = 1), then merged them with the original cohort and reperformed the multivariate logistic regression analysis for our primary conclusion. We found that even under this most conservative assumption, the shorter length of stay associated with the EVD+CD group that we previously identified remained statistically significant (p = 0.026).

We fully agree that this is an important methodological consideration. Following your suggestion, we have now more clearly elaborated the rationale for this exclusion criterion in the **Discussion section** of the revised manuscript and have included the corresponding statistical data for the additional patients (those who died or were lost to follow-up) **in the supplementary materials.**

We sincerely hope that the explanations provided and the supplementary analyses conducted will adequately address your concerns and demonstrate that our primary conclusions remain robust despite this methodological choice.

Once again, we deeply appreciate the time you have taken to provide your insightful comments, which have significantly enhanced the quality of our manuscript.

**Question 4:**

We fully acknowledge this as a definitive limitation of our study. As this was a retrospective analysis, some patients lacked either the baseline imaging within 24-48 hours post-aneurysm surgery or the systematic follow-up imaging at 3-6 weeks. Consequently, we were unable to re-evaluate all cases strictly according to the Vergouwen’s criteria. We sincerely regret this limitation.

Following your suggestion, we have explicitly added the statement to the **Limitations section of the Discussion** in the revised manuscript. We believe that the explanations provided and the corresponding revisions made to our manuscript adequately address your valuable comment. Once again, we extend our sincere gratitude for your insightful feedback, which has significantly enhanced the scientific rigor of our study.

**Question 5:**

We sincerely thank you for pointing out this important omission in our literature review. We fully agree that including and referencing previous studies on basal cistern catheters is crucial for properly contextualizing the background and significance of our research.

Following your suggestion, we have conducted a comprehensive search and review of contemporary research on basal cistern catheters, and have added the relevant references to the reference list of the revised manuscript（*Effectiveness of external ventricular drainage versus lumbar drainage in aneurysmal subarachnoid hemorrhage: A propensity-matched multicenter study; Nitric oxide metabolites in cisternal CSF correlate with cerebral vasospasm in patients with a subarachnoid haemorrhage; Enhanced cisternal drainage and cerebral vasospasm in early aneurysm surgery; Definition of Delayed Cerebral Ischemia After Aneurysmal Subarachnoid Hemorrhage as an Outcome Event in Clinical Trials and Observational Studies*）. We believe these additions now provide a more comprehensive and balanced view of the existing literature, properly contextualizing our findings within the broader scientific conversation. This significantly enhances the academic rigor of our paper.

**Response to Reviewer 2：**

We sincerely thank the reviewer for their positive feedback and valuable comments on our manuscript. We have carefully considered all the points raised and have revised the manuscript accordingly. The point-by-point responses are provided below.

**Question 1:**

We thank the reviewer for this excellent suggestion. We have now explicitly cited all figures at their appropriate first mention in the main text to improve the flow and readability for the reader.

**Question 2:**

We agree with the reviewer that providing the rationale for statistical test selection enhances methodological clarity.

In our experiment, non-continuous variables (such as cerebrospinal fluid cell count, cerebrospinal fluid protein levels, IL-6, IL-8, TNF-α, ET-1, MCP-1, and VCAM-1) are analyzed using repeated measures ANOVA. As for the ICP data analysis, a paired t-test was employed to observe whether there were significant differences between the two drainage methods.

**Question 3:**

We thank the reviewer for this prudent suggestion. We have revised the concluding paragraph of the manuscript to temper the language and explicitly state the need for future validation. Following your suggestion, we have explicitly added the statement to the **Discussion** section in the revised manuscript.
